# Supplementary material for: Near-infrared phosphorescent carbon dots for sonodynamic precision tumor therapy
Source: Nat Commun. 2022 Sep 30;13:5735. doi: 10.1038/s41467-022-33474-8 (PMC9523047; doi:10.1038/s41467-022-33474-8)
Supplement: Supplementary file 1 — Supplementary Information [file 41467_2022_33474_MOESM1_ESM.pdf]

Supplementary Information

Near-infrared phosphorescent carbon dots for sonodynamic precision tumor therapy

Bijiang Geng[1], Jinyan Hu[1], Yuan Li[2], Shini Feng[2], Dengyu Pan[1], Lingyan Feng[3], Longxiang Shen[4]

[1]School of Environmental and Chemical Engineering, Shanghai University, Shanghai 200444, China.

[2]School of Life Sciences, Shanghai University, Shanghai 200444, China.

[3]Materials Genome Institute, Shanghai University, Shanghai, 200444, China.

[4]Department of Orthopedic Surgery, Shanghai Jiao Tong University affiliated Sixth People's Hospital, Shanghai 200233, China.

Correspondence and requests for materials should be addressed to D.P. (e-mail: dypan617@shu.edu.cn) or to L.F. (lingyanfeng@t.shu.edu.cn) or to L.S. (e-mail: 7250012700@shsmu.edu.cn)

Contents

Supplementary Fig. 1 XRD characterization of p-CDs, n-CDs, and p-n-CDs.

Supplementary Fig. 2 Raman characterization of p-CDs, n-CDs, and p-n-CDs.

Supplementary Fig. 3 XPS characterization of p-n-CDs.

Supplementary Fig. 4 XPS characterization of p-CDs.

Supplementary Fig. 5 XPS characterization of n-CDs.

Supplementary Fig. 6 FTIR characterization of ICG, p-CDs, n-CDs, and p-n-CDs.

Supplementary Fig. 7 Zeta potential measurements of p-CDs, n-CDs, and p-n-CDs.

Supplementary Fig. 8 Colloidal stability evaluation of p-n-CD.

Supplementary Fig. 9 Electrochemical measurements of p-CDs, n-CDs, and p-n-CDs.

Supplementary Fig. 10 Optical characterization of ICG and p-n-CD.

Supplementary Fig. 11 Sonodynamic performance measurements of p-CD, n-CD, and p-n-CD.

Supplementary Fig. 12 Sonodynamic performance measurements of ICG and TiO_2.

Supplementary Fig. 13 Sonodynamic performance measurements.

Supplementary Fig. 14 Photodynamic performance measurements of p-n-CD.

Supplementary Fig. 15 Morphology characterization of p-n-CD@CCM.

Supplementary Fig. 16 Zeta potential measurements of p-n-CDs, CCM, and p-n-CD@CCM.

Supplementary Fig. 17 Colloidal stability evaluation of p-n-CD@CCM.

Supplementary Fig. 18 Sonodynamic performance measurements of p-n-CD@CCM.

Supplementary Fig. 19 Live/dead cell staining of 143B cells after different treatments.

Supplementary Fig. 20 ROS staining of 143B cells after different treatments.

Supplementary Fig. 21 Semi-quantitative analysis of live/dead cell staining and ROS staining.

Supplementary Fig. 22 Apoptosis assay of 143B cells after different treatments.

Supplementary Fig. 23 Quantitative analysis of in vivo NIR fluorescence images of p-n-CDs and p-n-CD@143B.

Supplementary Fig. 24 In vivo NIR fluorescence images of p-n-CD@Hela.

Supplementary Fig. 25 Ex vivo NIR fluorescence images of p-n-CD@Hela.

Supplementary Fig. 26 Quantitative analysis of in vivo NIR fluorescence images of p-n-CD@143B and p-n-CD@Hela.

Supplementary Fig. 27 Evaluation of tumor growth inhibition efficacy after different treatments.

Supplementary Fig. 28 Histological analysis of tumors after different treatments.

Supplementary Fig. 29 Evaluation of deep-tissue tumor therapeutic effect after different treatments.

Supplementary Fig. 30 Pharmacokinetic study of p-n-CD and p-n-CD@CCM.

Supplementary Fig. 31 Biodistribution study of p-n-CD@CCM.

Supplementary Fig. 32 In vivo biocompatibility studies by histological analysis.

Supplementary Fig. 33 In vivo biocompatibility studies by blood biochemistry and blood routine analysis.

Supplementary Fig. 34 Gating strategy for flow cytometry analysis.

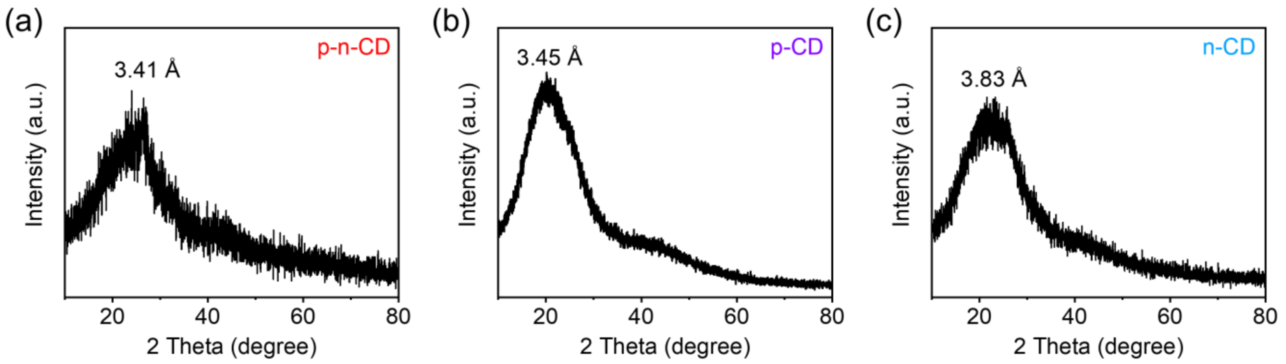

Supplementary Fig. 1 XRD characterization of p-CDs, n-CDs, and p-n-CDs. XRD patterns of p-n-CDs (a), p-CDs (b), and n-CDs (c). Source data are provided as a Source Data file.

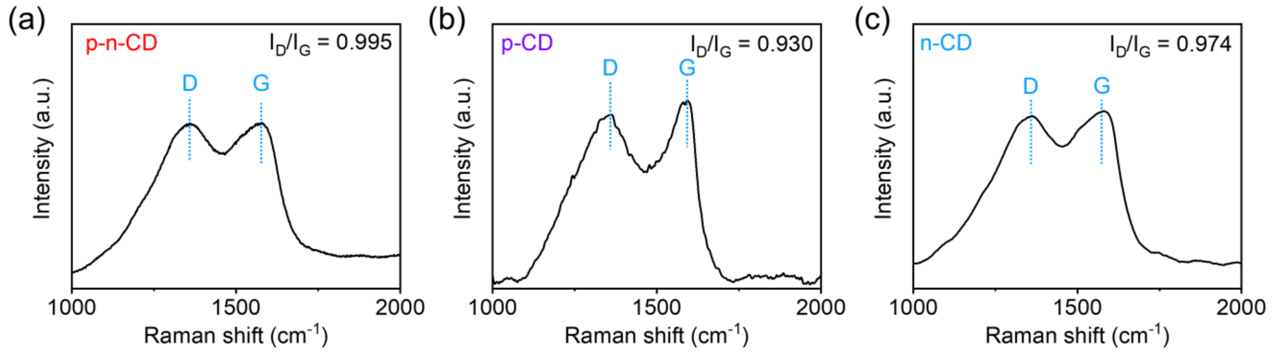

Supplementary Fig. 2 Raman characterization of p-CDs, n-CDs, and p-n-CDs. Raman spectra of p-n-CDs (a), p-CDs (b), and n-CDs (c). Source data are provided as a Source Data file.

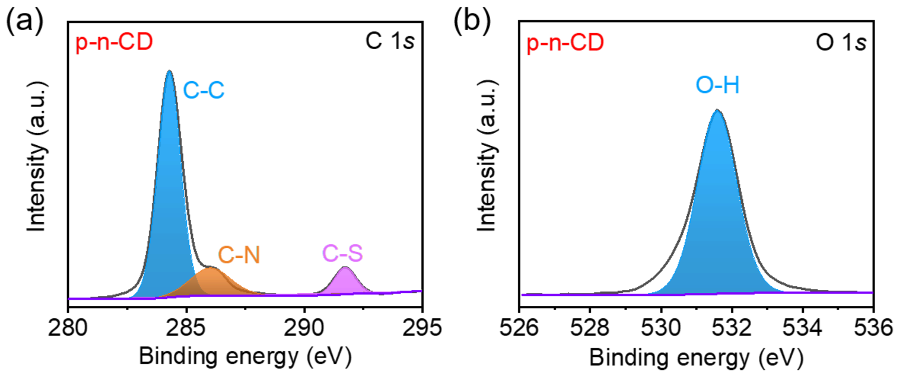

Supplementary Fig. 3 XPS characterization of p-n-CDs. High-resolution C 1s (a) and O 1s (b) spectra of p-n-CDs. Source data are provided as a Source Data file.

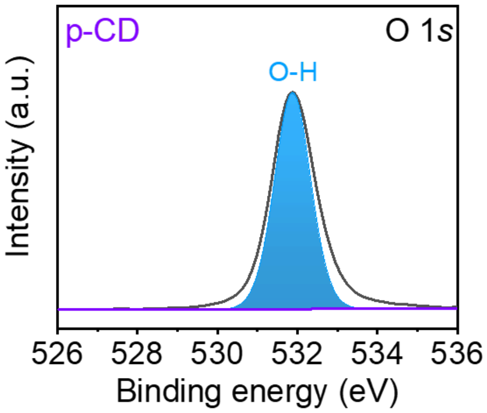

Supplementary Fig. 4 XPS characterization of p-CDs. High-resolution O 1s spectrum of p-CDs. Source data are provided as a Source Data file.

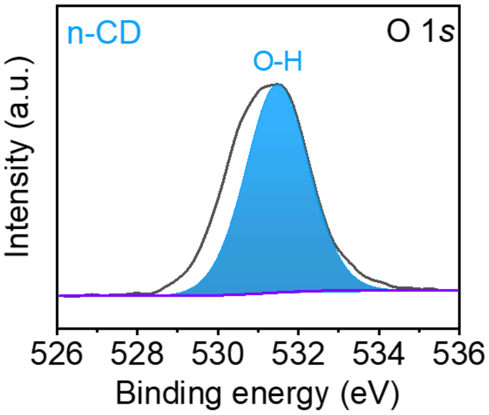

Supplementary Fig. 5 XPS characterization of n-CDs. High-resolution O 1s spectrum of n-CDs. Source data are provided as a Source Data file.

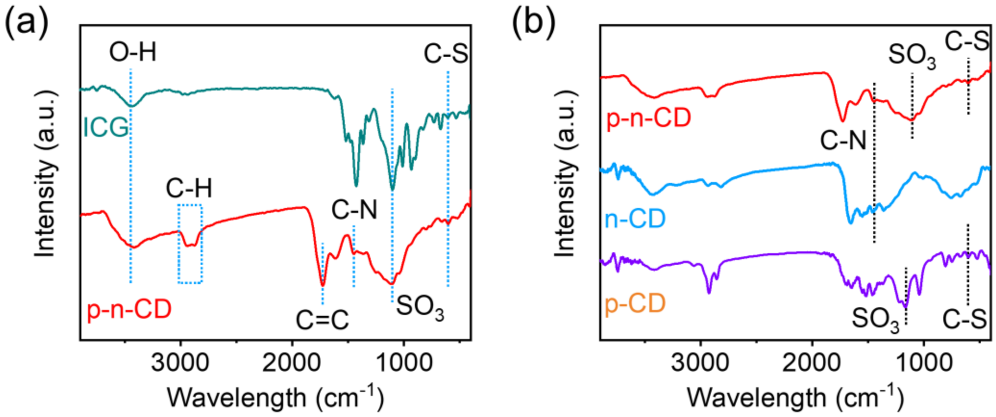

Supplementary Fig. 6 FTIR characterization of ICG, p-CDs, n-CDs, and p-n-CDs. FTIR spectra of ICG, p-n-CDs, p-CDs, and n-CDs. Source data are provided as a Source Data file.

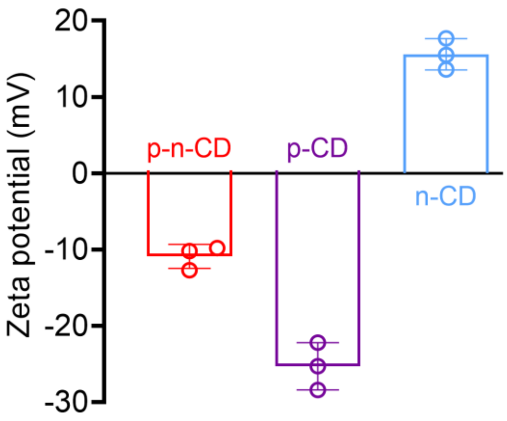

Supplementary Fig. 7 Zeta potential measurements of p-CDs, n-CDs, and p-n-CDs. Zeta potential of p-n-CDs, p-CDs, and n-CDs (n=3 biologically independent samples). Data are presented as mean values ± SD. Source data are provided as a Source Data file.

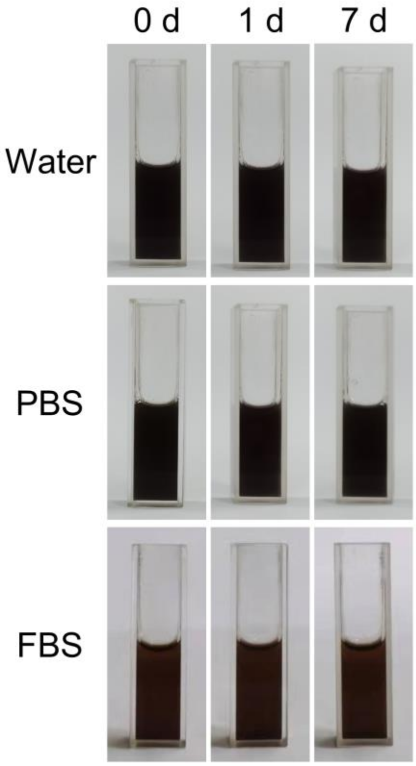

Supplementary Fig. 8 Colloidal stability evaluation of p-n-CD. Photographs of p-n-CD aqueous solution, PBS solution, and fetal bovine serum (FBS) solution stored for different periods of time (0, 1, and 7 days).

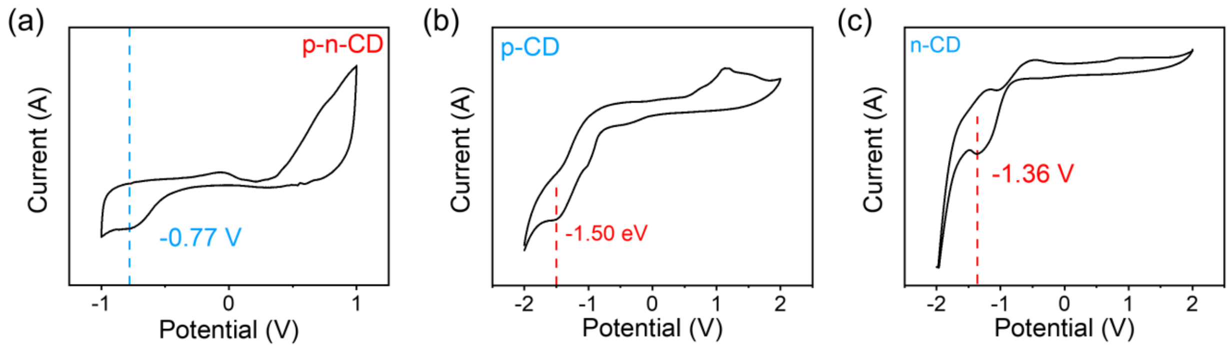

Supplementary Fig. 9 Electrochemical measurements of p-CDs, n-CDs, and p-n-CDs. CV curves of p-n-CDs (a), p-CDs (b), and n-CDs (c) with potentials vs Ag/AgCl. Source data are provided as a Source Data file.

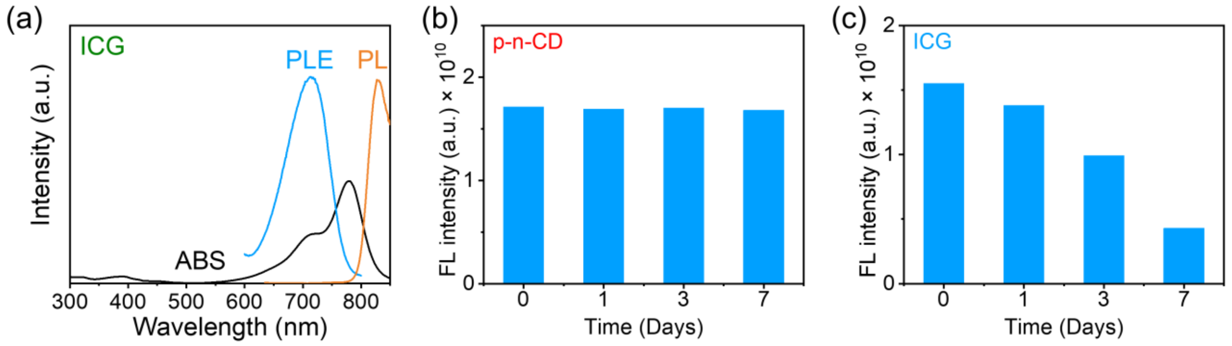

Supplementary Fig. 10 Optical characterization of ICG and p-n-CDs. (a) UV-vis-NIR absorption, PL, and PLE spectra of ICG. (b, c) NIR fluorescence signal intensity of p-n-CDs (b) and ICG (c) after storing for 0, 1, 3, and 7 days. Source data are provided as a Source Data file.

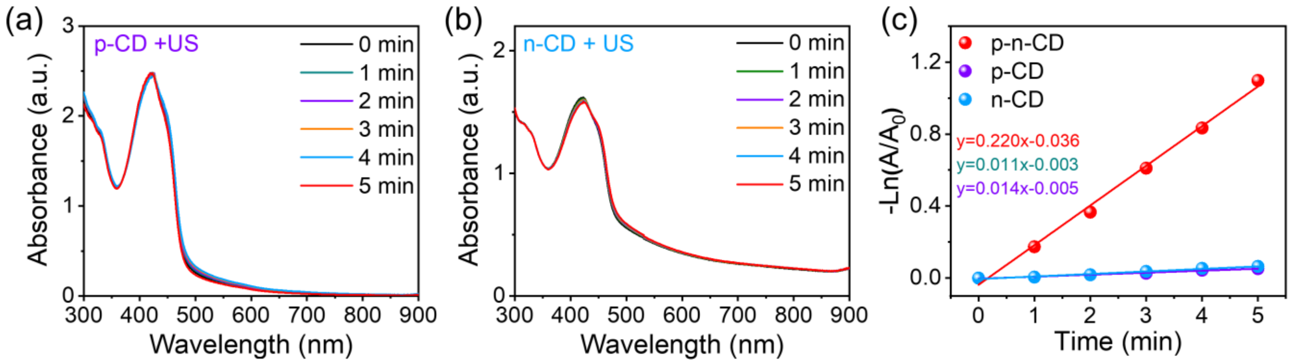

Supplementary Fig. 11 Sonodynamic performance measurements of p-CD, n-CD, and p-n-CDs. (a, b) Time-dependent $^1O_2$ generation by p-CDs (a) or n-CDs (b) under US irradiation (50 kHz, 3.0 W cm^{-2}). (c) The rate constant of $^1O_2$ generation in the presence of p-n-CDs, p-CDs, or n-CDs under US irradiation (50 kHz, 3.0 W cm^{-2}). Source data are provided as a Source Data file.

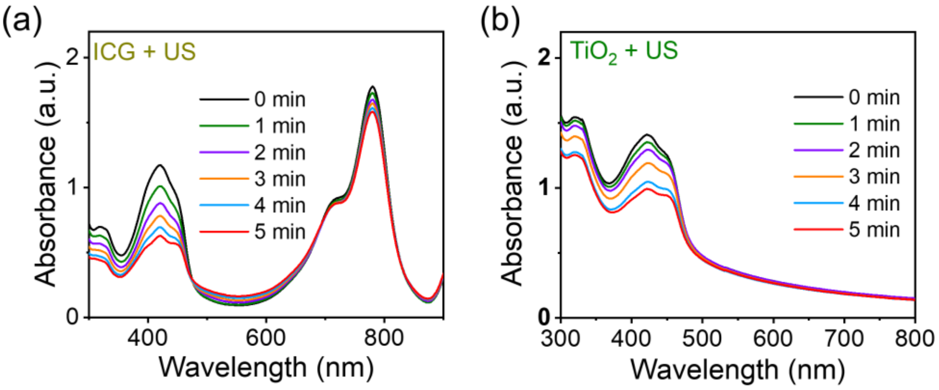

Supplementary Fig. 12 Sonodynamic performance measurements of ICG and TiO_2. Time-dependent $^1O_2$ generation by ICG (a) or commercial TiO_2 nanoparticles (b) under US irradiation (50 kHz, 3.0 W cm^{-2}). Source data are provided as a Source Data file.

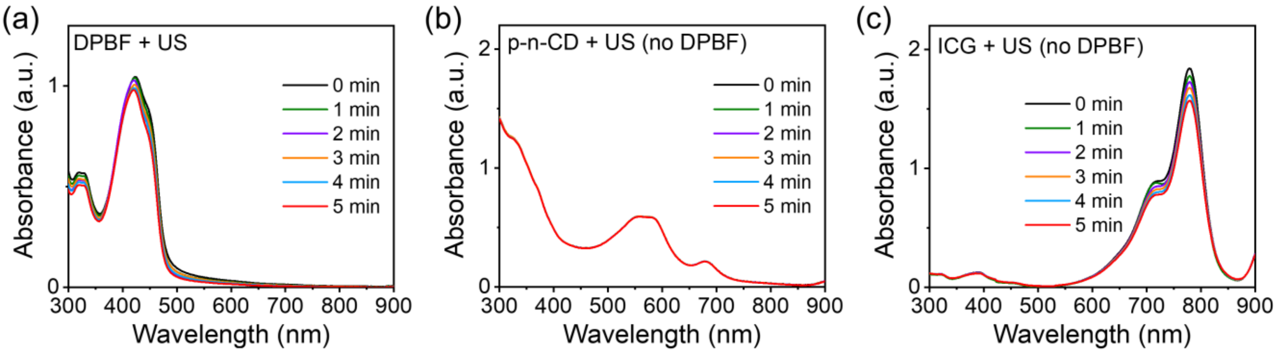

Supplementary Fig. 13 Sonodynamic performance measurements. Time-dependent $^1O_2$ generation by DPBF + US irradiation (a), p-n-CD + US irradiation (no DPBF) (b), or ICG + US irradiation (no DPBF) (c). Source data are provided as a Source Data file.

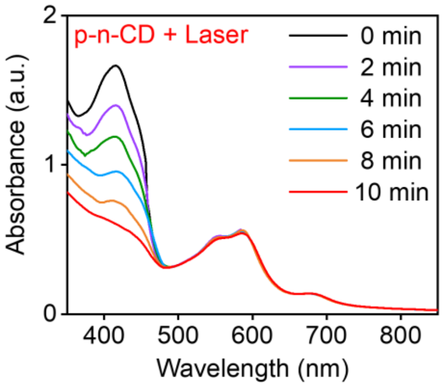

Supplementary Fig. 14 Photodynamic performance measurements of p-n-CDs. Time-dependent $^1O_2$ generation of p-n-CDs under 660 nm laser irradiation (0.2 W/cm^2) for 10 min detected with the DPBF probe. Source data are provided as a Source Data file.

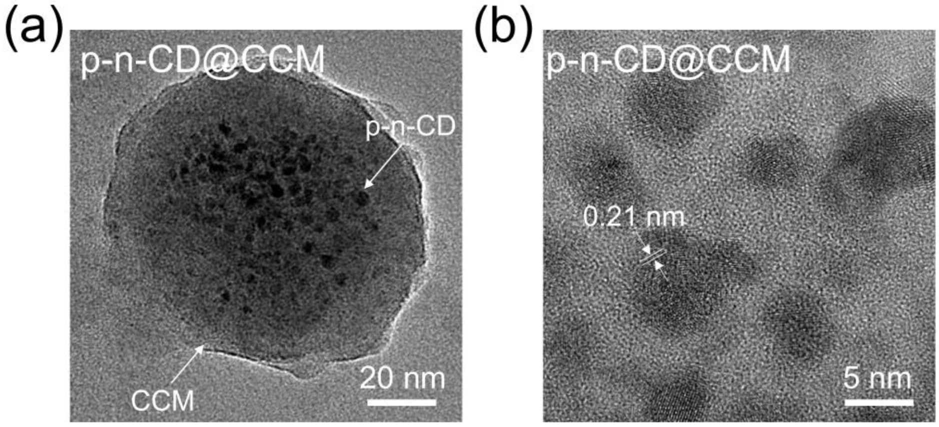

Supplementary Fig. 15 Morphology characterization of p-n-CD@CCM. TEM (a) and HRTEM (b) images of p-n-CD@CCM. A representative image of three biological replicates from each group is shown in (a), (b).

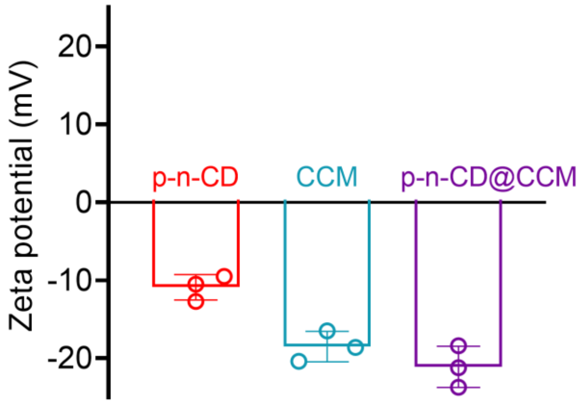

Supplementary Fig. 16 Zeta potential measurements of p-n-CDs, CCM, and p-n-CD@CCM. Zeta potential of p-n-CDs, CCM, and p-n-CD@CCM (n=3 biologically independent samples). Data are presented as mean values ± SD. Source data are provided as a Source Data file.

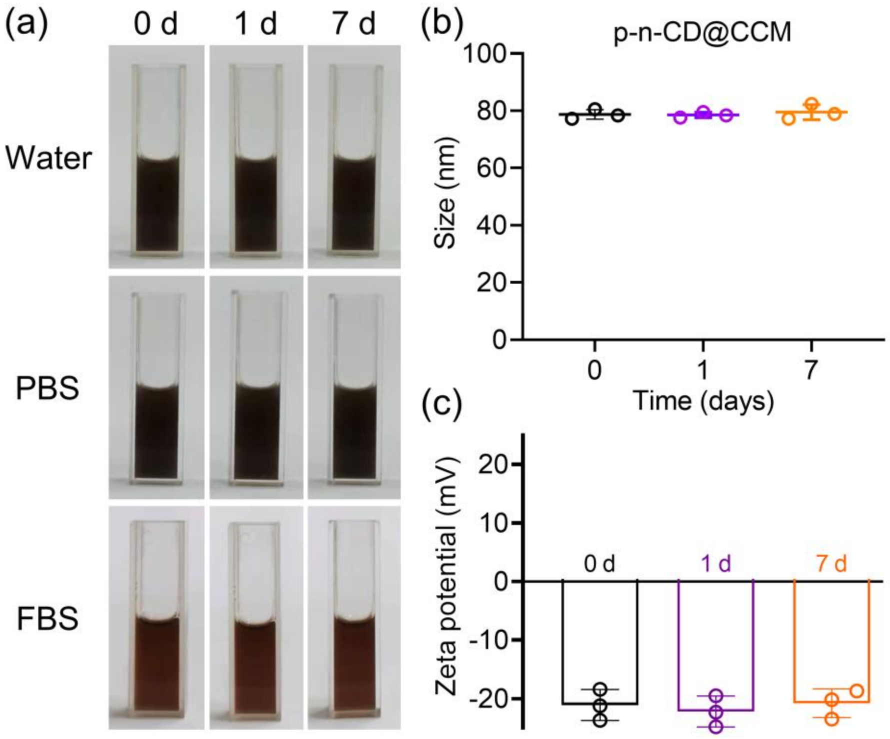

Supplementary Fig. 17 Colloidal stability evaluation of p-n-CD@CCM. (a) Photographs of p-n-CD@CCM aqueous solution, PBS solution, and fetal bovine serum (FBS) solution stored for different periods of time (0, 1, and 7 days). (b, c) Hydrodynamic diameters (b) and Zeta potential (c) of p-n-CD@CCM aqueous solution stored for different periods of time (0, 1, and 7 days) (n=3 biologically independent samples). Data are presented as mean values ± SD. Source data are provided as a Source Data file.

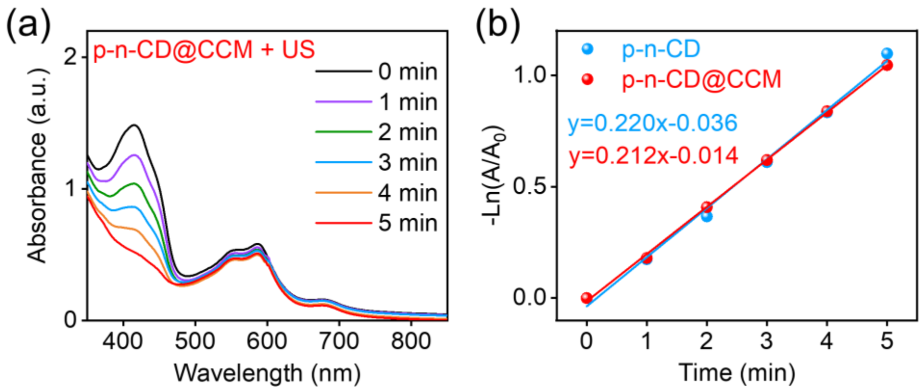

Supplementary Fig. 18 Sonodynamic performance measurements of p-n-CD@CCM. (a) Time-dependent $^1O_2$ generation of p-n-CD@CCM under US irradiation (50 kHz, 3.0 W cm^{-2}) for 5 min detected with the DPBF probe. (b) Rate constant of US triggered $^1O_2$ generation in the presence of p-n-CDs and p-n-CD@CCM under the same US irradiation. Source data are provided as a Source Data file.

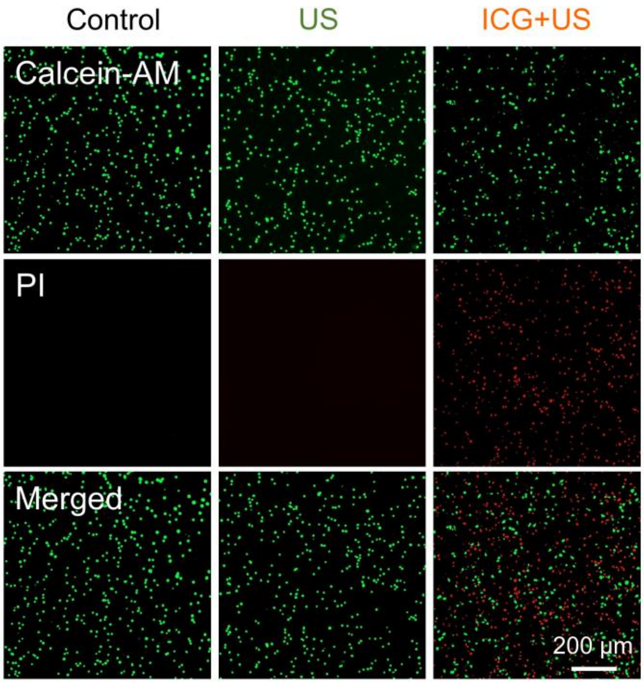

Supplementary Fig. 19 Live/dead cell staining of 143B cells after different treatments. Live/dead cell staining of 143B cells after different treatments including control, US alone, and ICG + US. A representative image of three biological replicates from each group is shown.

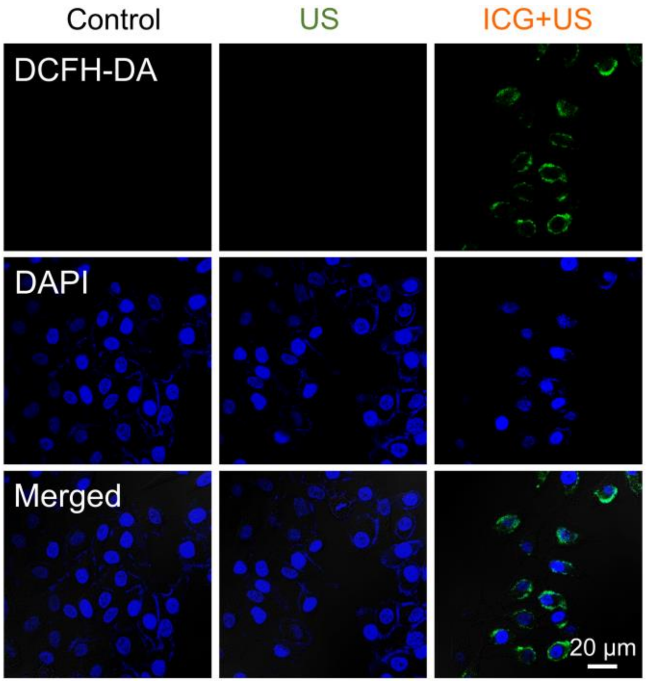

Supplementary Fig. 20 ROS staining of 143B cells after different treatments. ROS staining of 143B cells after different treatments including control, US alone, and ICG + US. A representative image of three biological replicates from each group is shown.

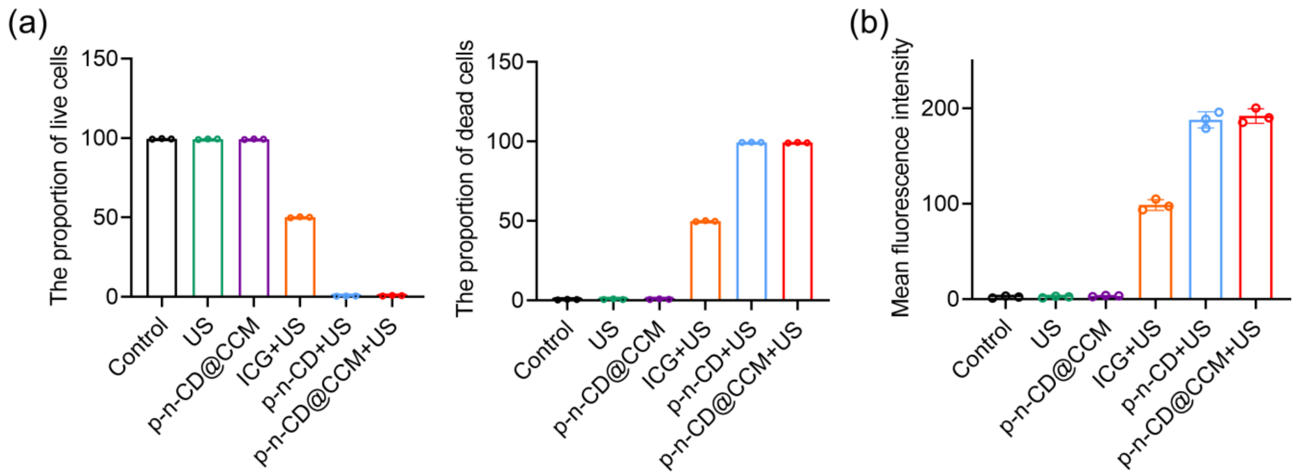

Supplementary Fig. 21 Semi-quantitative analysis of live/dead cell staining and ROS staining. (a) Semi-quantitative analysis of live/dead cell staining determined by the Image J software via triplicate parallel samples, indicating the proportion of live cells or dead cells in different groups (n=3 biologically independent samples). (b) Semi-quantitative analysis of ROS staining images determined by the Image J software via triplicate parallel samples (n=3 biologically independent samples). Data are presented as mean values ± SD. Source data are provided as a Source Data file.

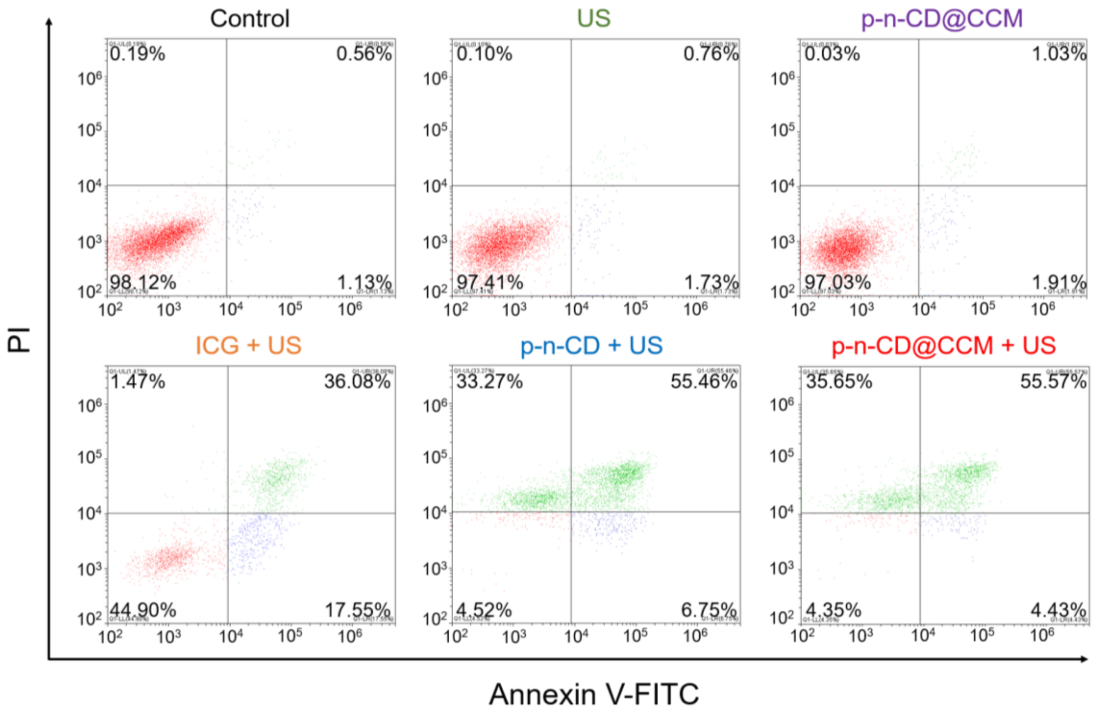

Supplementary Fig. 22 Apoptosis assay of 143B cells after different treatments. Flow cytometry apoptosis assay of 143B cells after different treatments including control, US alone, p-n-CD@CCM alone, ICG + US, p-n-CD + US, and p-n-CD@CCM + US.

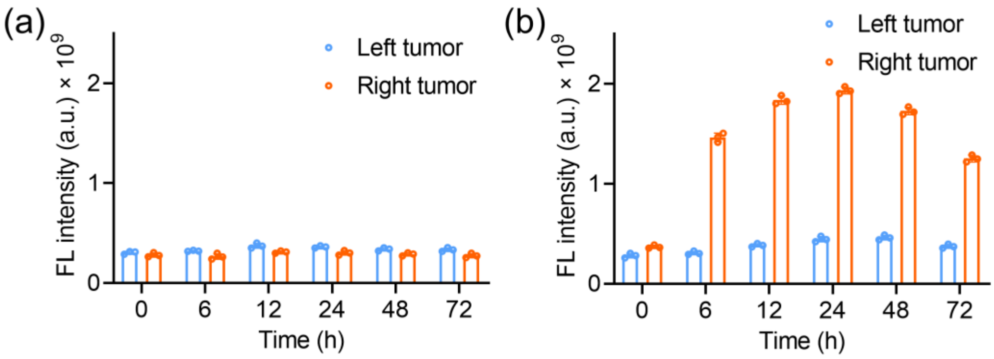

Supplementary Fig. 23 Quantitative analysis of in vivo NIR fluorescence images of p-n-CDs and p-n-CD@143B. NIR fluorescence signal intensity of the left tumor (Hela) and right tumor (143B) at 24 h post intravenous injection with p-n-CDs (a) or p-n-CD@143B (b) (n=3 biologically independent samples). Data are presented as mean values ± SD. Source data are provided as a Source Data file.

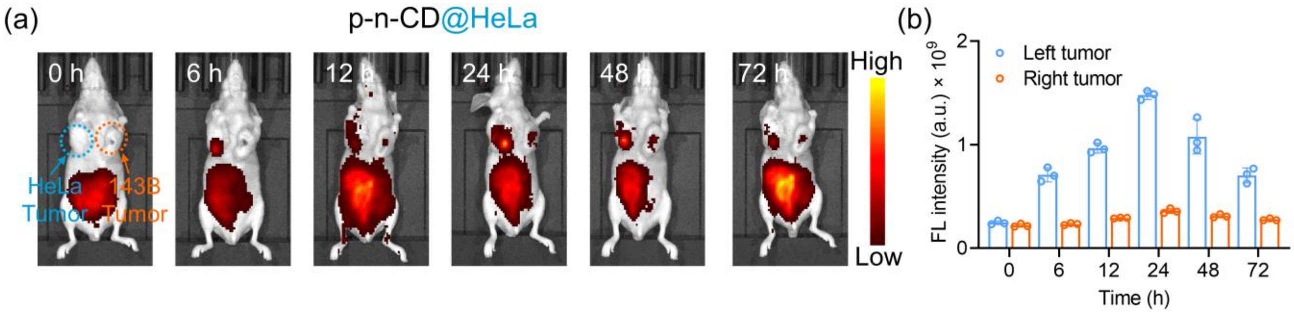

Supplementary Fig. 24 In vivo NIR fluorescence images of p-n-CD@Hela. (a) In vivo NIR fluorescence images at 24 h post intravenous injection with p-n-CD@Hela. (b) NIR fluorescence signal intensity of the left tumor (Hela) and right tumor (143B) at 24 h post intravenous injection with p-n-CD@Hela (n=3 biologically independent samples). Data are presented as mean values ± SD. Source data are provided as a Source Data file.

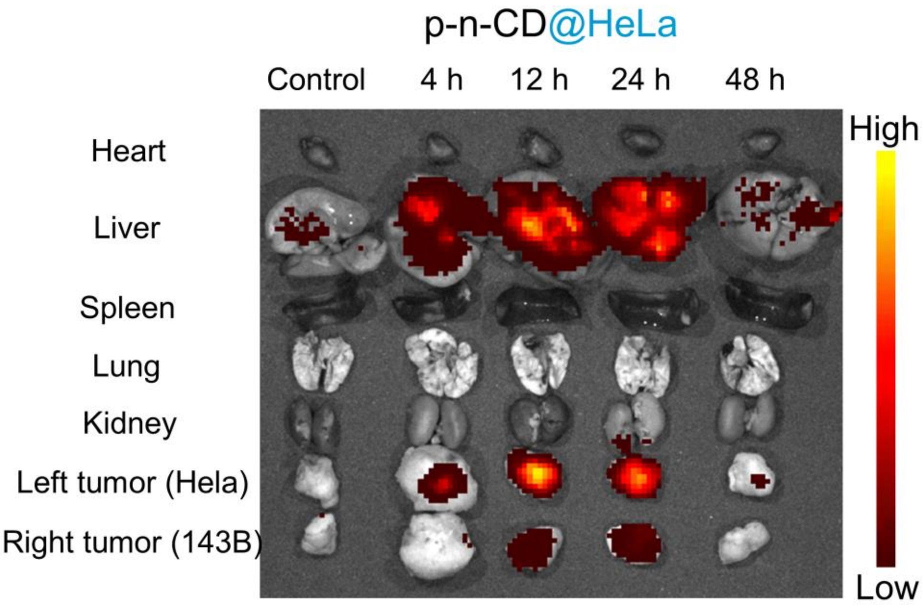

Supplementary Fig. 25 Ex vivo NIR fluorescence images of p-n-CD@Hela. Ex vivo NIR fluorescence images at 24 h post intravenous injection with p-n-CD@Hela.

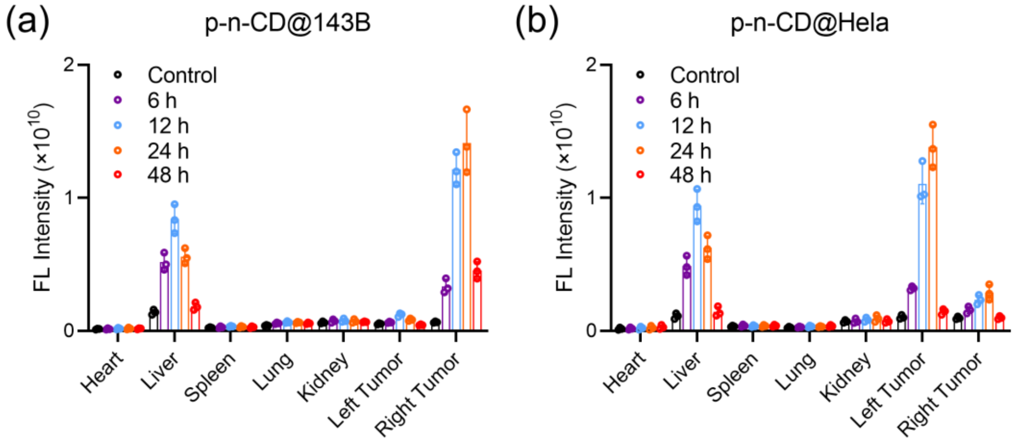

Supplementary Fig. 26 Quantitative analysis of in vivo NIR fluorescence images of p-n-CD@143B and p-n-CD@Hela. Time-dependent NIR fluorescence intensity of p-n-CD@143B (a) and p-n-CD@Hela (b) in the major organs and tumor tissues based on the fluorescence imaging results (n=3 biologically independent samples). Data are presented as mean values ± SD. Source data are provided as a Source Data file.

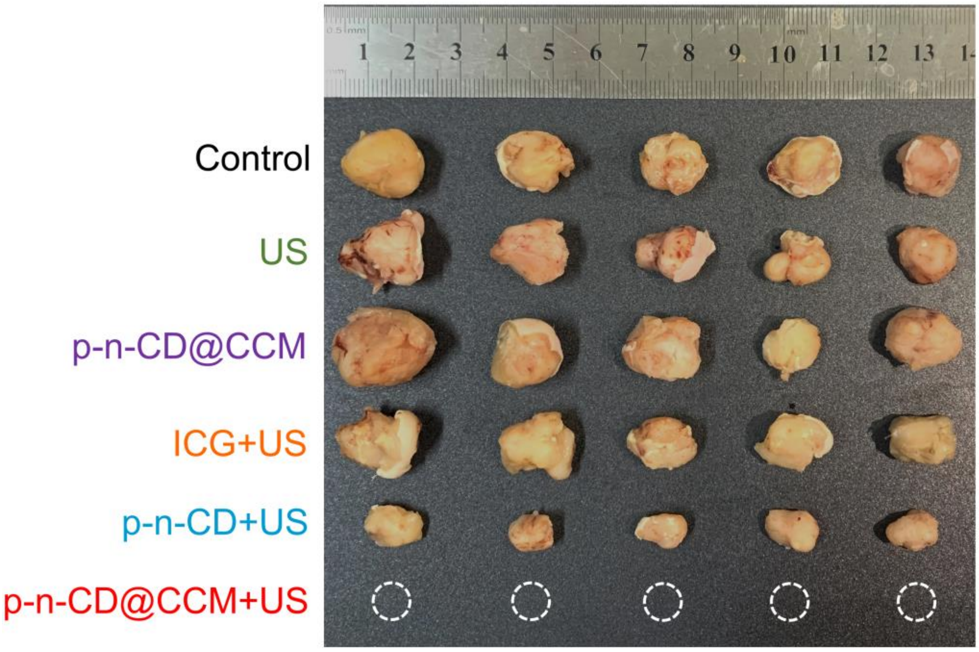

Supplementary Fig. 27 Evaluation of tumor growth inhibition efficacy after different treatments. Representative photographs of tumors after different treatments including control, US alone, p-n-CD@CCM alone, ICG + US, p-n-CD + US, and p-n-CD@CCM + US.

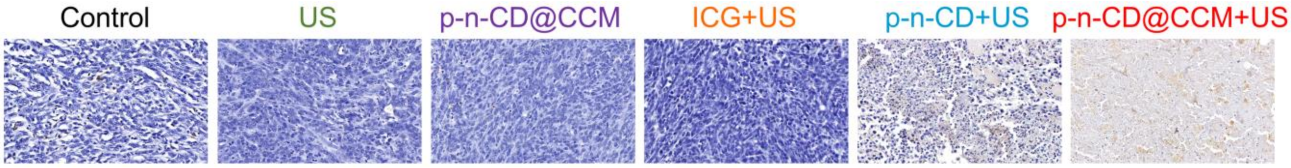

Supplementary Fig. 28 Histological analysis of tumors after different treatments. TUNEL staining of the tumors after different treatments. A representative image of three biological replicates from each group is shown.

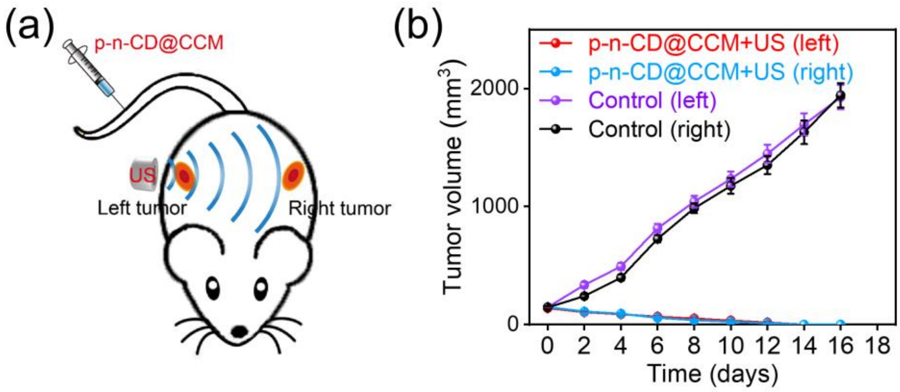

Supplementary Fig. 29 Evaluation of deep-tissue tumor therapeutic effect after different treatments. (a) Schematic illustration of p-n-CD@CCM treatments to 143B tumors in the left side and right side via the SDT. The US penetrated from left to right. (b) 143B tumor growth curves of mice

after indicated treatments (n=5 biologically independent samples). Data are presented as mean values ± SD. Source data are provided as a Source Data file.

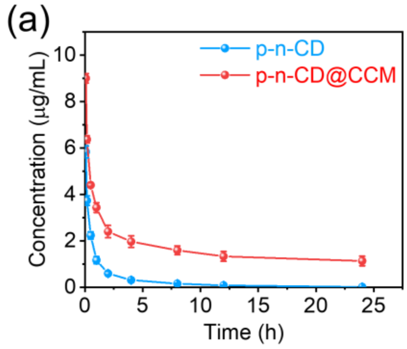

(b)

Parameters	p-n-CD	p-n-CD@CCM
C_{max} (μg/mL)	5.8±0.3	9.0±0.2
$t_{1/2}$ (h)	0.8±0.1	11.6±0.9
$AUC_{(o-\infty)}$ (μL•h/mL)	4.3±0.5	50.8±5.8
$MRT_{(o-\infty)}$ (h)	0.9±0.1	16.0±1.1
CL (L/h/kg)	0.5±0.1	0.04±0.006

Supplementary Fig. 30 Pharmacokinetic study of p-n-CD and p-n-CD@CCM. Plasma concentration-time profiles (a) and pharmacokinetic parameters (b) of p-n-CD and p-n-CD@CCM after intravenous administration of various drug formulations at the p-n-CD dose of 2.0 mg/kg (n=5 biologically independent samples). Data are presented as mean values ± SD. Source data are provided as a Source Data file.

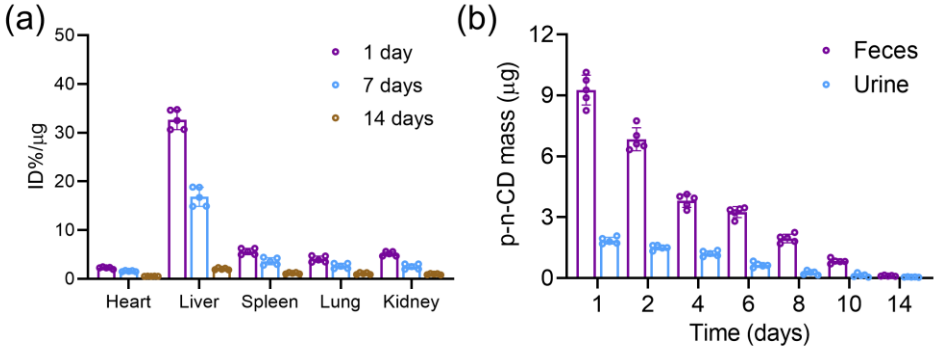

Supplementary Fig. 31 Biodistribution study of p-n-CD@CCM. (a) Biodistribution of p-n-CD@CCM post i.v. injection in mice on different days (n=5 biologically independent samples). (b) The detected p-n-CD mass in urine and feces at different time points post i.v. injection of p-n-CD@CCM (n=5 biologically independent samples). Data are presented as mean values ± SD. Source data are provided as a Source Data file.

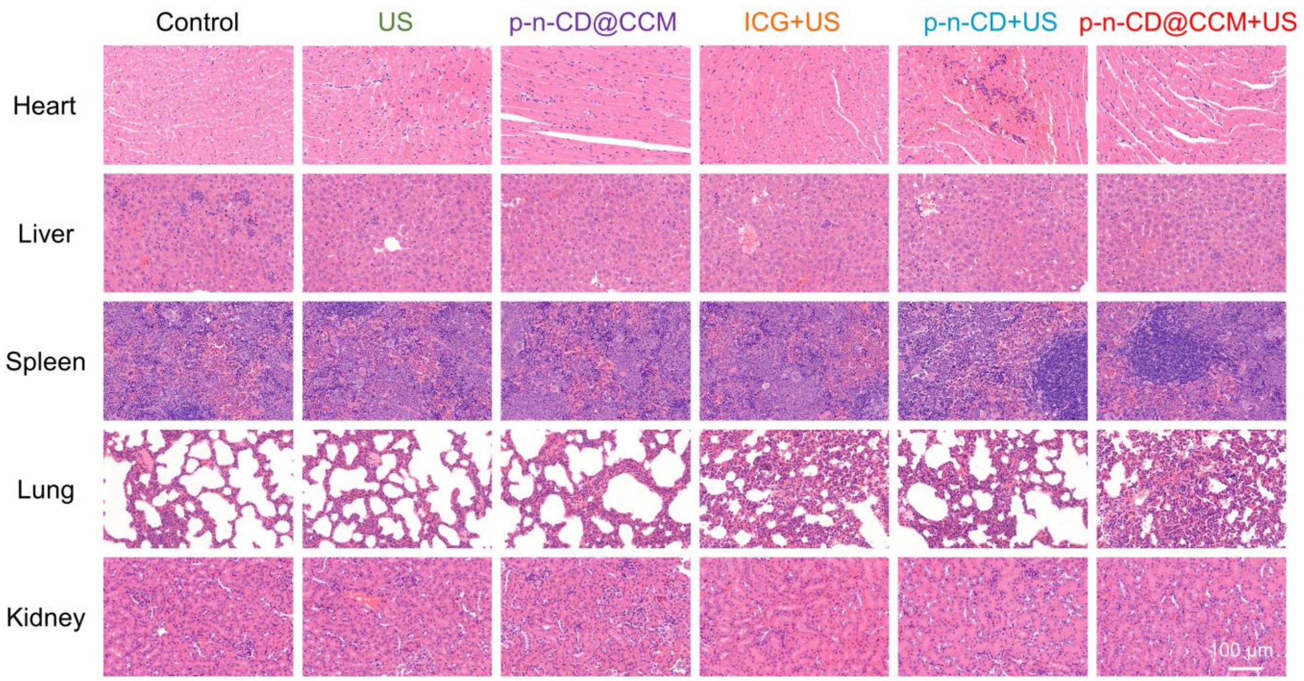

Supplementary Fig. 32 In vivo biocompatibility studies by histological analysis. H&E-stained images obtained from the major organs (heart, liver, spleen, lung, and kidney) of mice in different treatment groups. A representative image of three biological replicates from each group is shown.

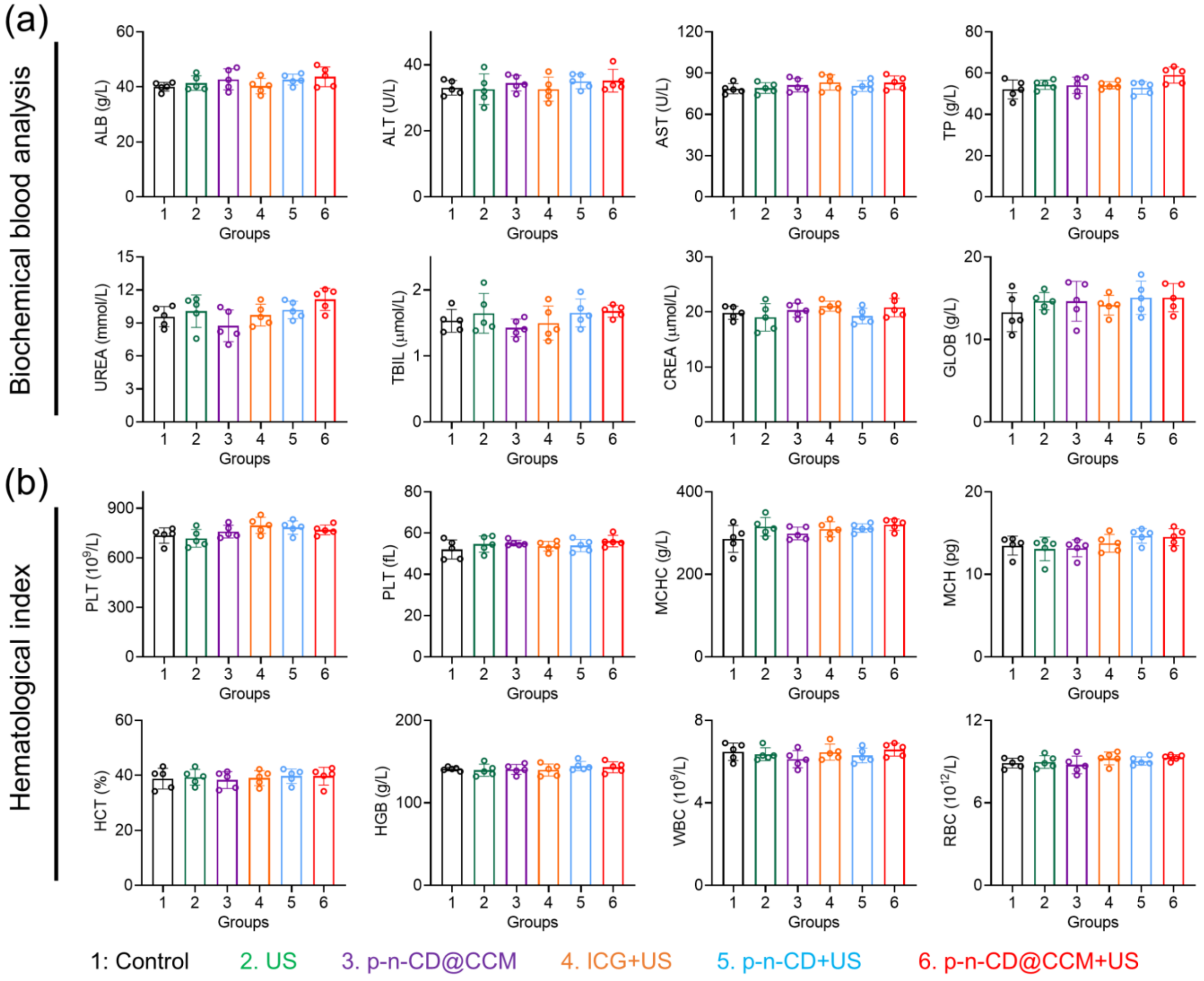

Supplementary Fig. 33 In vivo biocompatibility studies by blood biochemistry and blood routine analysis. (a-b) Biochemical blood analysis (a) and hematological index (b) of the mice that were sacrificed at 18 days after different treatments (n=5 biologically independent samples). The terms of biochemical blood analysis include ALB, ALT, AST, TP, UREA, TBIL, CREA, and GLOB. The terms of hematological index include PLT, MCV, MCHC, MCH, HCT, Hb, WBC, and RBC. Data are presented as mean values ± SD. Source data are provided as a Source Data file.

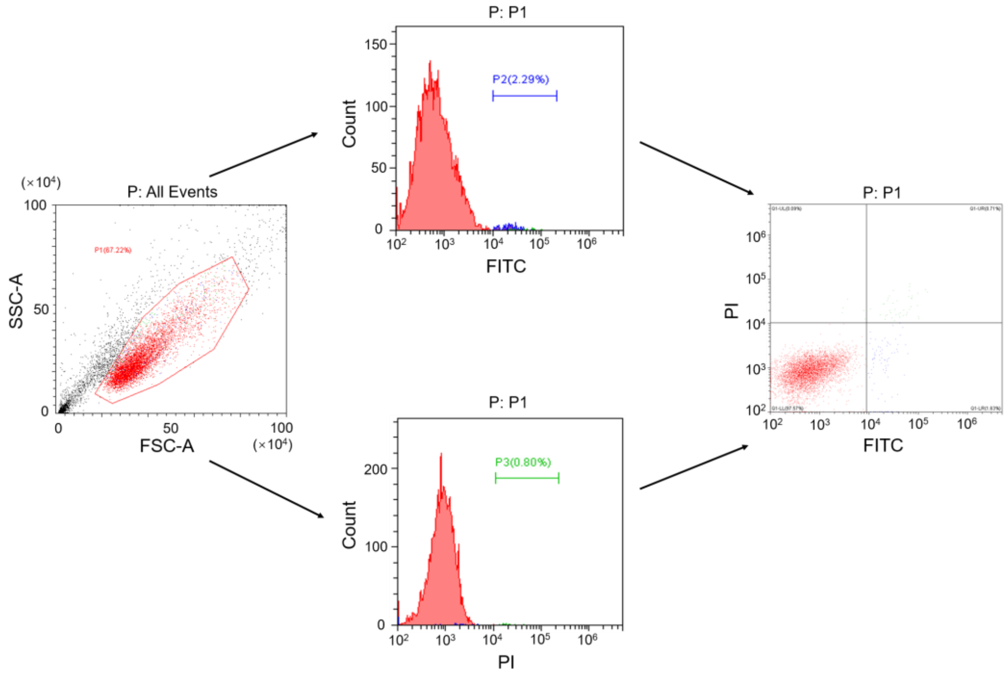

Supplementary Fig. 34 Gating strategy for flow cytometry analysis. Gating strategy for determining FITC and PI staining.
